# Elusive Diagnostic Markers for Russian Wheat Aphid Resistance in Bread Wheat: Deliberating and Reviewing the Status Quo

**DOI:** 10.3390/ijms21218271

**Published:** 2020-11-04

**Authors:** Vicki L. Tolmay, Scott L. Sydenham, Thandeka N. Sikhakhane, Bongiwe N. Nhlapho, Toi J. Tsilo

**Affiliations:** 1 Agricultural Research Council, Small Grain, Private Bag X29, Bethlehem 9700, South Africa; ssydenham@longreachpb.com.au (S.L.S.); tn.mboma@gmail.com (T.N.S.); bongiwe.nhlapho@yahoo.com (B.N.N.); tsilot@arc.agric.za (T.J.T.); 2 Department of Life and Consumer Sciences, University of South Africa, Pretoria 0002, South Africa

**Keywords:** *Diuraphis noxia* (Kurdjumov), host plant resistance, insect-resistance breeding, marker-assisted selection, *Triticum aestivum* L.

## Abstract

Russian wheat aphid, *Diuraphis noxia* (Kurdjumov), is a severe pest of wheat, *Triticum aestivum* L., throughout the world. Resistant cultivars are viewed as the most economical and environmentally viable control available. Studies to identify molecular markers to facilitate resistance breeding started in the 1990s, and still continue. This paper reviews and discusses the literature pertaining to the *D. noxia* R-genes on chromosome 7D, and markers reported to be associated with them. Individual plants with known phenotypes from a panel of South African wheat accessions are used as examples. Despite significant inputs from various research groups over many years, diagnostic markers for resistance to *D. noxia* remain elusive. Factors that may have impeded critical investigation, thus blurring the accumulation of a coherent body of information applicable to *Dn* resistance, are discussed. This review calls for a more fastidious approach to the interpretation of results, especially considering the growing evidence pointing to the complex regulation of aphid resistance response pathways in plants. Appropriate reflection on prior studies, together with emerging knowledge regarding the complexity and specificity of the *D. noxia*–wheat resistance interaction, should enable scientists to address the challenges of protecting wheat against this pest in future.

## 1. Introduction

The Russian wheat aphid (RWA; *Diuraphis noxia* (Kurdjumov), (Homoptera: Aphididae)) has been known as a severe pest of wheat (*Triticum aestivum* L.) and barley (*Hordeum vulgare* L.) since devastating losses were reported in the Crimea in 1901 [1], as quoted by [2]. This atypical grain aphid now appears throughout the world [3,4,5,6,7,8,9,10,11,12,13,14], following the 2016 report of its arrival in Australia [15]. Sometimes where *D. noxia* occurs, population levels remain below injurious levels. Damage is however regularly reported in areas characterized by medium-to-lower yield potentials, rain-fed conditions and sporadic droughts [16]. Several traits contribute to causing severe yield loss (60% or more) if this aphid is not controlled [17]. *D. noxia* infestation leads to dramatic chlorotic streaks on leaves [18], leaf-rolling, general stunting, and head-trapping [19] resulting in a sizeable loss of the photosynthetic area. Furthermore, *D. noxia* has a low developmental-threshold temperature [19], a vast host range throughout the grasses [20], and is protected from many generalist natural enemies by the rolled-leaf pseudo-gall it engenders [21]. Climate change and increased crop pest dispersal make finding tools for breeding resistant cultivars so as to control *D. noxia* more important than ever before.

In South Africa, *D. noxia* control was achieved using resistant cultivars, which formed the basis of an integrated pest management program [22]. Since 1992, >40 *Dn*-resistant (*Dn* after *D. noxia*) wheat cultivars have been released for cultivation. Research, started in 1978, successively focused on four South African *D. noxia* biotypes, namely RWASA1, RWASA2, RWASA3 and RWASA4, with an additional biotype, RWASA5, reported in 2019 [23]. These biotypes now occur concurrently in wheat-producing areas of the country [24,25,26,27]. Providentially, numerous sources of *Dn resistance* were rapidly identified following the incursions of *D. noxia* in both South Africa [28] and the United States [29]. On-going research [30,31,32,33,34,35,36,37,38,39,40,41,42,43,44,45,46,47,48,49,50,51,52,53] provided an ample number of accessions with genetic resistance to this pest. Numerous different mechanisms of resistance to *D. noxia* occur in these sources.

A 2016 review [54] concluded that, for aphid–plant interactions, multiple mechanisms could function at different stages of the interaction, and that these could differ for species pairs at different stages of co-evolution. Furthermore, the stealthy nature of aphid feeding in phloem makes these interactions highly distinct [54]. Within the known sources of *Dn resistance*, large variation occurs with respect to the mechanisms of resistance (antibiosis, antixenosis and tolerance [55]) that are expressed. Different plant metabolic processes of resistance were reviewed by [56]. Considerable evidence points to the role of phloem as a signaling network in addition to its primary role in the partitioning of photo-assimilates [57].

*Dn resistance*, which maintains chlorophyll functionality and thus yield under *D. noxia* infestation, was deployed in winter/facultative cultivars (Supplementary Figure S1 Map of South African wheat production regions) with considerable success and economic benefit [58]. Conventional back-cross-breeding, and the phenotypic screening of host plant resistance using bioassays with live aphids, was used to breed these cultivars [51]. Recently, marker-assisted selection (MAS) for *Dn resistance* breeding has been explored to facilitate gene/quantitative trait loci (QTL) stacking in order to achieve durable resistance to different pests/diseases or several biotypes of the same pest [59,60,61]. This could replace the phenotypic screening of plants [62,63] with a faster and higher throughput methodology.

Combining R-genes is no guarantee that resistance will be improved or more durable. There are arguments both for and against stacking aphid resistance genes in single accessions. For *Acyrthosiphon kondoi* Shinji (blue alfalfa aphid) resistance in *Medicago truncatula*, gene stacking enhanced aphid resistance with a complex interaction between genes in the pyramid [64,65]. The combination of R-genes *Rag4* and *Rag1b* against *Aphis glycines* (soybean aphid), however, resulted in very susceptible progeny [66]. Importantly, the durability of successful gene stacks is not yet predictable. Naturally occurring R-gene groups named ‘hot spots’ occur where genes that confer resistance to aphids, other insects and pathogens occur together [67]. Some aphid R-genes/QTL identified to date appear to be pleiotropic [68,69], and others epistatic [65,70]. Much research is needed to fully understand how R-gene combinations function. An alternate option to stacking R-genes is the use of mosaic-planting of crop cultivars with different R-genes. This production practice challenges pests with a complex genetic environment, which has been shown to decrease pest fitness [71,72,73].

### 1.1. Chronicling Marker Development for D. noxia Resistance

#### 1.1.1. Initial Studies

The search for *Dn resistance* markers in bread wheat began in South Africa and the USA in the early 1990s, using RAPD, RAPD-SCAR, PCR-RFLP and RFLP markers to explore donor landraces and near-isogenic lines [74,75,76,77,78]. As new marker technology developed, it was harnessed. By 2001, microsatellite/simple-sequence repeat (SSR) markers on chromosome 7D had been identified to tag *Dn resistance* genes. The gene *Dn2* was sub-divided into three “types” based on band size heterogeneity [79], while SSRs with specific size bands were reported to mark *Dn1*, *Dn2*, *Dn5(sic)*, *Dnx*, *Dn8* and *Dn9* [80]. Ambiguity ensued regarding the location and naming of *Dn5* following this paper [80,81]. New SSR markers on chromosome 1D for *Dn4* and *Dn6* [60] followed shortly, while a *Dn1* marker was confirmed [82], as cited by [83]. *Dn4* markers *Xgwm106* and *Xgwm337*, with estimated genetic distances of 7.4 and 12.9 cM [60], were confirmed in a second study with shorter linkage distances (5.9 and 9.2 cM, respectively) and slightly different band sizes using a different F_2:3_ population [84,85]. As with the 7D marker studies, variance between 1D marker studies caused confusion.

In 2005, the authors of [86] attempted to clarify the inconsistency in literature regarding the location and genetic relationships of the *Dn resistance* genes on 7D, namely *Dn1*, *Dn2*, *Dn5*, *Dn6* and *Dnx.* This study also included five additional donor accessions with uncharacterized *Dn*-genes. It concluded that the majority of *Dn*-genes on 7D are located on the 7DS arm, and that the genes appear either allelic or are tightly linked to one another in a *Dn*-gene cluster. A smaller resistance cluster was confirmed on chromosome 1DS [86] with *Dn4* [60] forming a part of this cluster. The position of *Dn5*, however, remained contested.

Monotelosomic 7DL plants carrying *Dn5* on the telosome were developed, and both the 7DS and 7DL telosomes were confirmed using mapped microsatellite and endopeptidase markers to show unequivocally that *Dn5* occurs on 7DL [87]. This 2006 study found an unknown *Dn*-gene, derived from the same donor as *Dn5*, i.e., PI 294994, on 7DS, substantiating the findings of a cluster on 7DS [86]. This *Dn resistance* gene on 7DS [80,87] has remained unnamed and is referred to in this paper as *DnUnknown*.

#### 1.1.2. Diverse Approaches to Dn resistance Marker Identification

Argentinian studies from 1999 onward focused on the identification and mapping of antibiosis and antixenosis to *D. noxia* [68,88]. A 2004 study [69] reported markers *Xpsr687* on 7DS and *Xgwm437* on 7DL for antixenosis, *Xpsr490* and *Rc3* on 7DS, and *Xgwm44*, *Xgwm437* and *Xgwm121* on 7DL for antibiosis, with at least two QTL in the repulsion phase, one near the centromere (7DS or 7DL) and the other distal on 7DL for antibiosis. In 2005, loci *Xgwm1293* and *Xgwm1150* on 6AL were associated with antixenosis against a new biotype present in Argentina [89].

By 2007, the research focus for *Dn resistance* markers shifted to genes effective against multiple *D. noxia* biotypes. Resistance breaking biotypes had, by that time, occurred in both the USA [90] and South Africa [25]. Markers were developed for the *Dn resistance* genes *Dn7* [91] and *Dn2414* [92]. However, both genes are associated with the “sticky dough” trait from the donor 1RS:1BL wheat-rye translocation. This regrettably made them unsuitable for use in bread wheat breeding programs.

Efforts from 2010 thus focused on the bread wheat accession, CItr 2401 (PI 9781), as it is also resistant to multiple *D. noxia* biotypes. A study [93] of a doubled haploid population identified numerous QTL associated with the foliar area (*Xpsp3103* on 4DS, and *Xgdm3* on 5DS), chlorophyll content (*Xgwm533* on 3BS and *Xpsp3094* on 7AL) and number of expanded leaves (*Xwmc264* on 3AS and *XwPt8836* on 4DS). Pleiotropic effects between the 4DS QTL and *Rht-D1* were noted, as were associations with orthologs of the markers [93]. Further scrutiny of CItr 2401 saw three papers [94,95,96] published, documenting the genetic basis of the *Dn240*1 resistance gene, which was mapped to 7DS. Four SSR markers, *Xcfd68*, *Xbarc214*, *Xgwm473* [94,96] and *Xcfd14* [96], and two single nucleotide polymorphisms (SNP), *Xowm705* and *Xowm711* [96], were identified closer to the *Dn2401* gene region through focused genetic studies. A 2019 *Dn2401* study [97] identified new SNP markers (*Xowm713*, *Xowm714*, *Xowm715* and *Xowm717*) to delineate a 0.3 cM and 133.2 kb interval which contains six high-confidence resistance gene candidates. Again, several credible studies have stimulated new questions.

Genome-wide association studies (GWAS) were also conducted to identify loci/chromosome regions that control *Dn resistance*. In 2013, an ICARDA study using 134 diverse wheat accessions [98] identified marker *wPt-733729* (7DS) associated with the leaf curling caused by *D. noxia*, as well as three markers, namely *wPt-3018* (7DL), *wPt-3291* (7DL) and *wPt-665471* (7DS), associated with leaf chlorosis. In 2016, Australian research identified new QTL for *Dn resistance* that mapped to chromosome 7DS [99]. This study hypothesized that the active area on 7DS, close to the centromere, is controlled by several loci, each providing small additive effects. These loci are tightly linked, segregate together, and may be a single locus comprising multiple alleles associated with specific phenotypes. A novel model was proposed suggesting that the *Dn*-genes at the 7DS locus are possibly contained within a chromatin loop [99].

Sadly, the markers reported above have not been properly validated in multiple wheat backgrounds, and the questions raised regarding pleiotropic effects and marker orthologs were never answered. To illustrate the enigmatic literature, five well-studied SSR markers are listed together with the reported band size for each linked *Dn*-gene/QTL (Table 1). The applicable *Dn resistance* donor accession used in each study, or the accession from which the study material was developed, is provided with the reference to the relevant study. It is prudent to note that all the *Dn*-genes mentioned in Table 1 (*Dn1*, *Dn2*, *Dn5*, *Dn6*, *Dn8*, *Dnx*, *DnUnknown*, *Dn2401* and *Dn626580*) are considered to occur on chromosome 7D near the centromere, but their exact position and how they interact with each other (i.e., alleles or part of an R-gene cluster) is still not yet entirely clear [49,60,80,86,87,95].

Other credible additional factors can be deduced from the literature in hindsight, and may shed light on significant aspects that could inadvertently have influenced this research field. The primary aim of this paper is thus to discuss the sometimes-contradictory literature pertaining to *Dn resistance* markers on chromosome 7D of wheat, and suggest plausible interpretations of the collective body of literature. Additional examples, obtained by testing some published SSR markers associated with *Dn resistance* on individual plants with known phenotypes from a panel of South African wheat accessions, will be presented. Prospective avenues for future research are alluded to, considering exciting current developments in the understanding of the complexities of the aphid–host plant resistance interaction.

## 2. Results

The mean phenotypic damage rating for the five example plants from each accession was used to rank them, from most resistant to least resistant to biotype RWASA2, and calculate the standard error of means, which is presented in Table 2 together with postulated potential genes in the accession.

### 2.1. Phenotyping

RWASA2, first reported as “Clone 2” [25], is virulent to *Dn1*, *Dn2*, *dn3*, *Dn8* and *Dn9* [27], while the genes *Dn4*, *Dn5*, *Dn6*, *Dn7*, *Dnx* and *Dny* remain effective against this biotype [27]. When considering the pedigrees of the accessions in the panel (see M&M), it is expected that several of them should be susceptible to RWASA2. This includes the susceptible control Hugenoot, as well as PI 137739“S” (*Dn1*), Betta-DN (*Dn1*), Gariep (*Dn1*), Tugela-DN (*Dn1*), PI 262660 (*Dn2*), BettaDn2, TugelaDn2, PI 634775 (*Dn8*), PI 634770 (*Dn9*) and T03/17 (*Dn1*, *Dn2*). The data confirm that, with the exception of PI 137739“S”, all the accessions one would expect to be susceptible to RWASA2 are indeed susceptible. There are, however, a number of accessions that are postulated to contain *D. noxia* resistance genes that should confer resistance to RWASA2, which are susceptible. It could thus be construed that T05/02 does not contain *Dn5*, Yumar does not contain *Dn4*, neither breeding-lines BW991306 nor BW991405 contain *Dn2401* or *Dn5*, and RIL-A50 does not contain *Dn2401*. PI 137739“S”, a selection from the original landrace PI 137739, must then contain either an additional gene to the reported *Dn1*, or a different gene that confers RWASA2 resistance. In addition to PI 137739“S” (*Dn137739”S”*), accessions CItr 2401 (*Dn2401*), T06/16 (*Dn5*, *Dn8*, *Dn9*, *DnUnknown*), PI 58654 (*Dnx*), PI 47545 (*Dn47545*), PAN 3144 (gene not known) and PI 626580 (*Dn626580*) tested as being resistant to RWASA2, with PI 586955 (*Dnx*), T06/13 (*Dn5*, *Dn8*, *Dn9*, *DnUnknown*), PI 243781 (*Dn6*) and PI 294,994 (*Dn5*, *Dn8*, *Dn9*, *DnUnknown*) testing as being moderately resistant to this biotype.

Notably, multiple *D. noxia* biotypes [27,100] occur concurrently in wheat fields in South Africa, although the predominant biotype may vary from season to season and within particular geographic regions. This requires that genes with resistance to different biotypes be combined within a single accession order to make multiple-biotype resistant cultivars available to producers. Due to the variation in resistance reactions present in different plants of the same accession, is not possible to stack *Dn resistance* against different biotypes without diagnostic molecular markers. A single plant can only be accurately phenotyped with one biotype in each generation. For example, a robust molecular marker for any gene(s) resistant to RWASA1 but susceptible to RWASA2 would enable breeders to combine the RWASA1-effective gene(s) with RWASA2-effective gene(s), for the control of more than one biotype concurrently. Screening the germplasm with RWASA2 would identify plants with RWASA2-effective resistance, and RWASA1-effective resistance could be identified by selecting those RWASA2-resistant plants that also contain the marker. The reciprocate is not possible, as most genes effective against RWASA2 would mask the presence of genes effective against RWASA1 (Personal communication, data not shown VL Tolmay) if the plants were phenotyped with the RWASA1 biotype. This seeming anomaly could easily be explained if the particular *Dn resistance* in these accessions is complex in nature, and is contingent on the *D. noxia* biotype used to develop/select the accession, with other biotypes either recognizing the whole or only parts of the complex resistance.

### 2.2. Genotyping

Of the five markers tested on this panel of accessions, *Xgwm473* and *Xgwm635* did not reflect sufficient polymorphism, and the data are therefore not shown. *Xgwm473* was reported to be linked to *Dn* resistance by two studies [49,95], with both groups describing a 244 bp fragment as the diagnostic band. However, the genes reportedly linked to this fragment were different, namely, *Dn626580* [49] and *Dn2401* [95]. *Xgwm635* was reported to be linked to *Dn8* from PI 294994 [80] with a 100 bp band. The three remaining markers, namely *Xgwm44*, *Xgwm111* and *Xgwm437*, for which PIC values [101] were calculated from the panel data (Supplementary Table S1), will be discussed below in the order they occur on the wheat consensus map [102] of chromosome 7D. It is, however, prudent to note that markers *Xgwm44* and *Xgwm111* have multiple orthologs, as reported [86,103] (Supplementary Table S1), potentially compounding allelic interpretations.

SSR marker *Xgwm44*, located on 7DS [80,86,104], is reported to give a 180 bp band for resistance gene *Dn6* from accessions PI 243781 [60,86] and PI 262660 (sic) [86], while resistance from accession PI 047545 was linked to a 200 bp fragment [86]. A 180 bp fragment was also reported for this marker for *DnUnknown* [87]. In this study, 12 haplotype combinations (Supplementary Table S1) of band sizes 0, 120, 130, 150, 175, 185, 190 and 200 bp were found in both individual resistant and susceptible plants.

Wheat microsatellite marker *Xgwm111*, on the short arm of chromosome 7D [86], has been associated with *Dn resistance* since the report [80] that it is tightly linked to *Dn1*, *Dn2*, *Dn5*(sic) and *Dnx*. The single band sizes reported for each of the genes in this study [80] were 210 bp [PI 137739], 200 bp [PI 262660], 220 bp [PI 294994] and 225 bp [PI220127], respectively. The resistance gene *Dn5*(sic) from PI 294994 identified by [80] is probably not the same as *Dn5*, named by and allocated to chromosome 7DL through a telosomic analysis [81]. A follow-up study [87] using *Xgwm437* placed *Dn5* on 7DL, as it was only amplified in 7DL monotelosomic plants. This corroborates the prior mapping [102,104] of *Xgwm437* on 7DL. Furthermore, the landrace PI 294994 is known to contain several different resistance variants [105,106]. The most widely accepted explanation for the confusion regarding resistance genes from this landrace is that the resistant plants used in these and other studies [80,81,105,106], though all linked to landrace PI 294994, differ from each other because different single plants were selected for use. The biotype used for the phenotypic evaluation of the plant reaction could be an additional factor contributing variability to the results, as the contradictory genetic studies identifying *Dn5* used different *D. noxia* biotypes, namely RWASA1 [81,87] and RWA1 [80], to evaluate for susceptible and resistant plants. Furthermore, the close proximity of the *D. noxia* R-genes to the centromere of chromosome 7D significantly affects recombination frequencies and further hinders clarity [87]. The literature reports *Xgwm111* band sizes 200, 210, 215, 220, 225 and 274 bp associated with the phenotypic expression of *Dn resistance* (Table 1). In this study, more allelic variation was observed with band sizes 0, 130, 135, 150, 180, 190, 200, 210 and 220 bp recorded in 16 haplotype combinations (Supplementary Table S1) from both resistant and susceptible plants. None of the plants in this study gave a band size of 215 bp [87], 225 bp [80] or 274 bp [95], despite the donor accessions PI 294994 and CItr 2401, in addition to numerous accessions developed from these accessions, present in the test panel.

Three different ‘types’ of bands were found with marker *Xgwm437*, located on 7DL [79], that were associated with resistant plants derived from accession PI 262660 (Table 1). These fragments were reported as the ‘highest bands’, and the illustration provided in this manuscript clearly shows multiple bands obtained with this marker. These three ‘highest bands’ with band sizes 104 bp (Type I), 102 bp (Type II) and 100 bp (Type III) are very close in size to the 105 bp band reported for *Dn5* [87] from PI 294994. A 105 bp fragment was also reported to be associated with *Dn626580* [49]. In this study, each of the 11 haplotypes (Supplementary Table S1) contained a single band of either 0, 90, 95, 100, 105, 110, 115, 120, 125, 130 or 135 bp for this panel. These haplotypes appear to occur in specific combinations with the haplotypes associated with *Xgwm111* and *Xgwm44*, alluding to the existence of a diverse resistance cluster or a block of allelic variants.

### 2.3. Correlation of Phenotype and Marker Results

To practically illustrate selection, using a combination of the phenotype resistance expression of one *D. noxia* biotype (in this case, RWASA2) and SSR markers for resistance to another biotype (for arguments sake, RWASA1), let us consider some examples (Table 3). Accessions derived from single R-gene-sources will be briefly discussed, before moving to those with potential combinations of R-genes from multiple sources.

Commercial cultivars Betta-DN, Gariep and Tugela-DN (all derived from PI 137739 and potentially containing *Dn1)* tested as being susceptible to RWASA2. They all contained the *Xgwm44*_120;190_ and *Xgwm111*_135;210_ haplotypes, while Betta-DN contained *Xgwm437*_120_, and both Gariep and Tugela-DN contained *Xgwm437*_115_ (Table 3). Advanced breeding-lines BettaDn2 and TugelaDn2 (derived from PI 262660 and potentially containing *Dn2*) were also susceptible to RWASA2. Within the 10 plants representing these two advanced breeding-lines, 9 plants contained the *Xgwm44*_120;150;190_ haplotype with 1 TugelaDn2 plant containing *Xgwm44*_null_. All five BettaDn2 plants as well as two of the TugelaDn2 plants contained haplotype *Xgwm111*_135;210_ as well as *Xgwm437*_100_. The remaining three TugelaDn2 plants contained *Xgwm111*_135;220_ and *Xgwm437*_115_. It is not unequivocally possible to confirm the presence of *Dn1* or *Dn2* based on these marker alleles, although the phenotypic data using RWASA2 is expected for plants containing these genes.

Both T05/02 plants and two of the T16/03 plants (derived from PI 294994 using RWASA1) tested as being susceptible to RWASA2, suggesting that they do not contain *Dn5*, although they may well contain *Dn8*, *Dn9* and *DnUnknown*, or any combination of the latter. The remaining two plants of T06/13 were resistant (a damage rating score of 6 or less) to RWASA2, and contained the *Xgwm44*_120;175_, *Xgwm111*_135;200_ and *Xgwm437*_120_ haplotypes. The susceptible plants contained different haplotypes, namely *Xgwm44*_null_, *Xgwm111*_135;210_ and *Xgwm437*_100_ (1 T05/03 plant); *Xgwm44*_120;175_, *Xgwm111*_135;200_ and *Xgwm437*_100_ (1 T05/03 plant) or *Xgwm44*_120;185_, *Xgwm111*_135;200_ and *Xgwm437*_120_ (2 T06/03 plants). Again, based on the above data, it is not possible to definitely confirm *Dn5* present in the two RWASA2-resistant plants. These individual plants cannot be rescreened using RWASA1 or any other biotype, and there is no guarantee that their progeny or other seeds from the same mother plant will have the same haplotypes as these individual plants.

Advanced breeding-lines T03/17 (*Dn1* and *Dn2*) and T06/16 (*Dn1* and *Dn5*, *Dn8*, *Dn9*, *DnUnknown*) were purposefully developed to combine *Dn*-genes from multiple sources. The haplotypes of the five T03/17 plants are *Xgwm44*_120;175_, *Xgwm111*_130;200_ and *Xgwm437*_120_, and all are susceptible to RWASA2. Similar to the reasoning for accessions containing either *Dn1* or *Dn2*, these results do not confirm the presence of either genes, nor whether the attempt to combine them was successful. According to the pedigree, advanced breeding-line T06/16 could potentially contain *Dn1*, *Dn5*, *Dn8*, *Dn9* and *DnUnknown*, or any combination of these genes. All five plants of this accession tested as being resistant when screened with RWASA2, phenotypically substantiating the postulated presence of *Dn5* as the only one of these genes reported to confer resistance to RWASA2. Haplotypes *Xgwm44*_120;175_, *Xgwm111*_130;200_ and *Xgwm437*_135_ occur in four plants with a higher level of resistance than the fifth, which contains marker haplotype *Xgwm44*_120;190_ instead of the 120; 175 bp band recorded for the other single plants. Again, the marker haplotypes do not correspond with the published information for *Dn5* or *DnUnknown*, and it is not clear whether *Dn1* is present in these plants at all. Had these plants been screened with RWASA1, the presence of *Dn5* (which was substantiated in this case by the RWASA2 screening that took place) would have masked the presence of *Dn1*, as both genes confer resistance to RWASA1.

The phenotypic reaction of resistant commercial cultivar and the check accession of PAN 3144 (see M&M) shows that it contains gene(s) conferring resistance to RWASA1, RWASA2 and RWASA3. Biotype characterization studies [27,100] list *Dn5*, *Dn6*, *Dn7* and *Dnx* as the only genes that confer resistance to all three of these biotypes. The haplotypes of the five resistant plants, namely *Xgwm44*_120;190 or 195_, *Xgwm111*_135;200 or 210_ and *Xgwm437*_120_, do not clearly indicate the presence of any of those genes in these plants.

Single plant data pertaining to the landrace R-donors and other accessions included in this study can be found in Supplementary Table S1. The five plants of landrace PI 137739”S” are uniform in terms of their genotype, as are those of PI 262660. In all likelihood, this is due to a specific, targeted selection in the case of PI 137739”S”, where a single plant with resistance to biotype RWASA3 = 5 was selected in January 2015. PI 262660 may inadvertently have become more uniform over many years of successive use and seed multiplication. Three haplotypes are present in the five plants of PI 294994, with only two plants, of the same haplotype, testing resistant to RWASA2. All four plants of landrace PI 047545 tested resistant to RWASA2, with the most resistant plant possessing a different haplotype to the others. Two haplotypes were also contained in the landrace PI 243781, with the single resistant plant different to the other susceptible ones, while in PI 626580 two haplotypes are present, but the most susceptible plant has the same haplotype as one of the most resistant. All three plants of CItr 2401 were resistant and contained the same haplotype. It would appear that the allelic diversity in landrace accessions may be dependent on whether the accession is still an amalgamation (bulked-up as collected) or whether a selection has been purified from it. Furthermore, allelic variation may be dependent on, or restricted by, which *D. noxia* biotype was used to characterize the accession or make the selection. The potential influence of the biotype used during the screening and selection would furthermore naturally affect/influence the robustness of the marker allele’s association with the trait. Generally, the haplotype data for the individual plants of single R-gene cultivars and advanced breeding-line accessions are sufficiently uniform to indicate that the accessions are true breeding. In the case of accessions developed to combine genes, the variation is somewhat greater.

## 3. Discussion

Many authors make an important distinction between markers useful for MAS and those that are not [103,107,108,109]. Generally, three critical requirements distinguish markers considered functional or diagnostic for MAS [107,110]. These are reliability, repeatability and robustness. The first requires flanking markers or tight (≤1 cm) linkage between the marker and the target gene/trait [108], as a larger distance can result in false selection [76,108]. The second is the validation of the marker–trait association across multiple genetic backgrounds [108,111], and the third is the suitability for large scale commercial application [108,109] versus the gain per unit time and cost [103]. The examples presented in this paper indicate that the markers tested did not meet the required level of reliability and repeatability across a panel of resistant and susceptible South African accessions. This is possibly due to the relatively large linkage distances and a lack of conclusive validation studies. In general, these examples show that multiple haplotypes exist in many of the test accessions, even when the phenotype is similar, and the accession is of an advanced enough generation to expect a true breeding response.

Two inter-related sources of ambiguity can be identified, which could account for the observed phenomena. Firstly, in terms of the host plant genetics, the dominant inheritance of most *Dn resistance*, often attributed to single genes [32,38,41,42,47,48,49,52,56,81,105,106,112,113,114,115], has underpinned many studies, despite evidence of a more complex genetic control of resistance [16,40,53,60,68,69,80,86,89,98,99,116]. ‘Downstream’ support for the last-mentioned hypothesis includes studies reporting multiple resistance mechanisms present in specific accessions [40,117,118,119], QTL associated with specific mechanisms [68,69,88,89,93,99], and studies reporting differential gene expression [120,121,122,123]. Additional compelling results include a 2009 paper [118] reporting multiple loci of genetic control within a single accession. Breeding-line KS94H871, containing *Dnx* from PI 220127, was shown to contain two loci encoding resistance to RWA1, but only one locus encoding resistance to RWA2. This pattern is echoed in the 2016 GWAS of a DH population (ECA Gregory x PI 94365) from Australia [99], with two loci (on 7D and 1D) encoding resistance to RWASA1 and RWASA2, while only the 1D locus encodes resistance to RWASA3. The re-evaluation and selection of resistant plants from ‘Plant Introduction’ accessions following the discovery of biotype USA2 in the United States [53] could point to mixed landrace accessions, as stated by the authors, but could alternately be explained by the “*Dn*-biotype-specific–R-gene(s)” concept shown in the aforementioned studies [99,118].

The second source of confusion could result from the *D. noxia* biotype used for the phenotypic evaluation of wheat accessions used in specific studies. This is true for the evaluation during the development of the test population and/or the evaluation of the phenotype which is used for trait-association analysis. In some instances, the biotype(s) used for the initial development of study-accessions and association mapping studies are not the same, while in some they are. The biotype is rarely specified per “*Dn*-marker–R-gene association”. Furthermore, *Dn* markers have rarely been validated using different/multiple biotypes to assess the specificity of the marker–trait association. Table 4 contains a summary of germplasms utilized in 7D marker studies, listing the *D. noxia* biotype(s) used to develop the accessions and evaluate the phenotype for genotypic association/marker development.

Inconsistencies with respect to fragment size between different studies, over many years in many wheat accessions, indicate that the marker alleles are not diagnostic. This may be potential *Dn*-gene allelic variation that has gone unresolved or un-noticed in the past. Nevertheless, the same markers are repeatedly found to be linked to *Dn resistance* on chromosome 7D. This indicates that genetic resistance to this pest is coded within those regions in some way. The current shortage of diagnostic markers for this trait should be addressed, taking account of the growing evidence for the complex regulation of resistance gene expression [126,127].

Across multiple crops, the complexity of aphid–plant interactions is being progressively revealed [71]. Despite multiple *D. noxia* biotype studies [128,129,130,131,132,133,134], many unknowns still have to be clarified. In general, “research shows that aphid virulence may be a complex adaptation involving a myriad of factors, including epigenetically controlled phenotypic plasticity and contributions from endosymbionts, the gut and saliva” [126]. Likewise, studies of plant defence against insects reveal that resistance gene expression and defence metabolism is influenced by both exogenous and endogenous environmental factors [71]. New evidence shows that plants utilize sophisticated mechanisms to modulate their response to stressors [135]. Embracing these unknowns within the current knowledge base [136], and engaging with them by using the ever-improving understanding of plant defence against insects, may lead to what has eluded us thus far. It is imperative that breeders are enabled with diagnostic markers with which to address the challenges posed by not only the insect pests, but also the changing climatic conditions which will undoubtedly influence pest distribution and the extent of damage they cause. The tools we need to breed *D. noxia*-resistant wheat will probably be based on a far better understanding of the specific *D. noxia*–host plant interactions. Two of the current developments in wheat to follow closely involve studies applying advanced molecular technologies to pinpoint *D. noxia* resistance genes [96,97] and to understand the regulation of the resistance response pathway [127,137,138]. Genetic characterization of the various donor sources of R-gene(s)/QTL, an understanding of the functional plant metabolism encoded by each genetic component, as well as a clear understanding of how these components interact with each other and the specific *D. noxia* biotype, will be essential to harnessing this plant-resistance to protect wheat in future.

## 4. Materials and Methods

### 4.1. Plant Materials

The 26 accession panel (Table 5) used to provide single plant examples in this study is comprised predominantly of wheat cultivars and advanced breeding-lines from the Agricultural Research Council-Small Grain (ARC-SG) *D. noxia* pre-breeding program, South Africa [139]. Based on pedigree data and phenotypic evaluation with multiple biotypes, the accessions are postulated to potentially contain different *Dn*-genes (Table 5) or combinations thereof. The wheat cultivars Gariep, Yumar and PAN 3144 are considered differential checks, and their different RWASA-biotype responses are shown in Table 6 together with those of the susceptible (Hugenoot) and resistant (CItr 2401) controls.

RWASA2 was chosen to phenotype the individual plants for marker validation. It is sufficiently damaging to allow discrimination, and all checks (Table 6) give consistent responses to it, while with other resistance breaking biotypes (RWASA3, RWASA4), a measure of segregation is known to occur. The reaction of accessions to the original South African biotype (Supplementary Table S2) was considered the baseline reaction of each accession.

### 4.2. Phenotypic Screening and Tissue Collection from Single Example Plants

A 21-day seedling assay [16] was performed to phenotype the test plants. In total, 15 individual seeds of each accession were planted in Professional Potting Mix^®^ (Cultera, Muldersdrift, South Africa, www.cultera.co.za). Five cones per accession containing three seeds were arranged in a randomized complete block design within two 98-cone trays and then watered with KynoPop™ (Kynoch, Sandton, South Africa, www.kynoch.co.za) seedling fertilizer. Seven days post-planting, fresh leaf tissue material for DNA extraction purposes was harvested from a single plant per cone for each accession, and the other plants that germinated within that cone were uprooted and discarded. Every accession was left with five individual plants that were then each infested with c. five individuals of apterous mixed instars of *D. noxia* biotype RWASA2. The RWASA2 biotype used in this study was obtained from a colony maintained at ARC-SG. The individual plants were scored 21 days post-infestation using a damage rating scale of 1–10, where 1 = Highly resistant and 10 = Dead [15].

### 4.3. DNA Isolation and Polymerase Chain Reaction (PCR)

The fresh leaf material, harvested from five individual plants of each test accession, was individually homogenized within 750 µL of extraction buffer for 1 min at 30 r/s with the Qiagen TissueLyser II. A modified cetyltrimethylammonium bromide (CTAB) DNA extraction protocol [146] was used to isolate genomic DNA, which was then treated with 2 µL RNase-A enzyme (Inqaba Biotechnical Industries (Pty) Ltd., Pretoria, South Africa). A Nanodrop 2000 Spectrophotometer (Thermo Scientific (Pty) Ltd., Waltham, MA, USA) was used to determine the quality, purity and concentration of each sample at the absorbance ratio of 260/280 nm. DNA samples were diluted with 1x TE (Tris-EDTA) buffer to 50 ng µL^−1^ final concentration and stored at 4 °C before progressing to downstream PCR applications. Five SSR marker primer pairs for *D. noxia* resistance, which occur on chromosome 7D, vis*. Xgwm44* [60,80], *Xgwm111* [60,80], *Xgwm437* [79], *Xgwm473* [49] and *Xgwm635* [80], were synthesized by Integrated DNA Technologies (Integrated DNA Technologies, Inc. Coralville, Iowa, USA, www.IDTDNA.com) and were provided by Whitehead Scientific PTY (Ltd) Cape Town, South Africa (www.whitesci.co.za). PCR reaction conditions recommended for the KAPA 2X Ready Mix PCR Kit (KAPA Biosystems, Cape Town, South Africa, www.kapabiosystems.com) were applied. Each PCR reaction consisted of 10 µL (1x) KAPATaq 2X Ready Mix, 0.5 µL (10 µM) per SSR primer and the remaining volume (5.0 µL) of DNAse-free water. PCR was performed with a profile comprising initial denaturation at 95 °C for 4 min, followed by 35 cycles of denaturation involving 95 °C for 30 s, annealing at a specific temperature for individual marker for 30 s, and extension at 72 °C for 30 s. Thereafter, a final extension step of 5 min at 72 °C was performed.

Relevant SSR marker-specific PCR amplicons were separated on 3.0–3.5% (*w*/*v*) high resolution agarose gel (Certified Low Range Ultra Agarose, Bio-Rad Laboratories, Inc. Hercules, CA, USA) stained with GelStar™ Nucleic Acid Gel stain (Lonza, Morristown, NJ, USA). Fragment separation was performed in an electrophoresis chamber containing 1x Tris-borate-EDTA (TBE) buffer and run at 100–125 V for 1–4 h. The SSR product sizes were determined according to 100 bp and/or 20 bp DNA ladders (Lonza SimplyLoad R, Lonza, Morristown, NJ, USA). A digital photograph was taken of the gel under UV light exposure with the Bio-Rad Molecular Imager Gel DocTM XR Instrument. Observed SSR marker alleles were sized, recorded and analyzed per cultivar both visually and with Bio-Rad image LabTM gel analysis software (Bio-Rad Laboratories, Inc., Hercules, CA, USA).

Data for each single example plant (damage rating score and successful marker analysis) are tabled in Supplementary Table S2. The mean phenotypic damage rating for the five single plants from each accession was used to rank the accessions from most resistant to least resistant, and calculate the standard error of means presented in Table 3.

## 5. Conclusions

In recent years, the spread of agricultural crop pests has become broader [147,148]. Prediction models [149] estimate that by the middle of this century, many important crop-producing countries will be fully saturated with pests. These authors [149] further state that, in spite of the quarantine and phytosanitary measures that are designed to prevent pest spread, natural dispersal and trade eventually result in invasions of crop pest species into previously pest-free areas. The global redistribution of species is not limited to pests that spread to previously pest-free areas. Virulent biotypes of pests can similarly spread, causing the resurgence of a pest in an area where it was formerly controlled. Climate change will undoubtedly influence the distribution and pest status of *D. noxia.* A clear understanding of the genetic control of *Dn resistance*, together with robust diagnostic markers, will be important in addressing challenges posed by this aphid in a timely manner.

The landrace origins and proximity of *Dn resistance* gene(s) to the centromere of 7D have been put forward as possible explanations for the difficulties encountered in the search for diagnostic markers for this trait to date. However, following thorough deliberation, it appears that additionally, two inadvertent faults may have blurred the accumulation of a coherent body of information applicable to *Dn resistance*. The “single dominant gene” assessment, initially accepted as the model of genetic control for resistance to this pest, has paradoxically permeated and simplified the underlying assumptions of many studies. This may have hindered critical investigation, despite multiple studies contending that *Dn resistance* is controlled by closely linked genes, multiple alleles at the same locus, or QTL influenced by the genetic background they occur in. Reconsideration of inadvertent assumptions or omissions, with appropriate reflection on the *D. noxia* biotype used to generate the data, may help better understand previous studies and plan future ones. This review calls for a more fastidious approach to the interpretation of results. Should it hold true that the genetic control of *D. noxia* resistance is more complex than originally thought, it could follow that a *D. noxia* biotype-specific R-gene/allele/QTL interaction, or possibly even a *D. noxia* biotype-specific resistance–response pathway interaction, may be at play. This, together with the potential pleiotropic and epistatic effects of genes involved in *Dn resistance*, should be investigated in future studies.

## Figures and Tables

**Table 1:** Five *D. noxia* resistance-linked markers and reported fragment sizes/additive effects for different *Dn*-genes/QTL from specific donor accessions on chromosome 7D of bread wheat.

Marker	Fragment Size (bp)/QTL Additive Effect	*D. noxia* R-Gene	Donor/(Test Accession(s))	Reference
*Xgwm44*	Four fragments between 80–182	None	(Chinese Spring, Thatcher) #	[60,86]
*Xgwm44*	185	None	(Chinese Spring)	[87]
*Xgwm44*	180	*DnUnknown*	PI 294994	[87]
*Xgwm44*	180	*Dn6*	PI 243781	[60]
*Xgwm44*	180	*Dn6*	PI 262660(sic)	[60]
*Xgwm44*	200	*Dn6*	PI 047545	[60]
*Xgwm44*	Instar duration −0.797 ** Aphid fertility −3.940 ** Longevity −13.457 ***	*QTL*	Doubled-Haploid Recombinant population of CS and 7D Synthetic	[69]
*Xgwm111*	Three fragments between 130–305	None	(Chinese Spring, Thatcher) #	[60]
*Xgwm111*	209	None	(Chinese Spring)	[87]
*Xgwm111*	200	*Dn2*	PI 262660	[80]
*Xgwm111*	200	*Dn6*	PI 243781	[60]
*Xgwm111*	210	*Dn1*	PI 137739	[87]
*Xgwm111*	210	Not yet named	PI 047545	[60]
*Xgwm111*	215	*DnUnknown*	PI 294994	[87]
*Xgwm111*	220	*Dn5*	PI 294994	[87]
*Xgwm111*	225	*Dnx*	PI 220127	[86]
*Xgwm111*	274	*Dn2401*	CItr 2401	[95]
*Xgwm111*	210, 240, 250	*Dn1*	PI 137739	[83]
*Xgwm111*	210, 240, 250	*Dn5*	PI 294994	[83]
*Xgwm111*	210, 240, 250	*None*	(Chinese Spring 7DS dt)	[83]
*Xgwm437*	112	*None*	Chinese Spring	[87]
*Xgwm437*	100 (Type III)	*Dn2*	PI 262660	[79]
*Xgwm437*	102 (Type II)	*Dn2*	PI 262660	[79]
*Xgwm437*	104 (Type I)	*Dn2*	PI 262660	[79]
*Xgwm437*	105	*Dn5*	PI 294994	[87]
*Xgwm437*	124	*Dn626580*	PI 626580	[49]
*Xgwm437*	Antixenosis +2.077 ** Longevity −27.420 ***	*QTL*	Doubled-Haploid Recombinant population of CS and 7D Synthetic	[69]
*Xgwm473*	244	*Dn626580*	PI 626580	[49]
*Xgwm473*	244	*Dn2401*	CItr 2401	[95]
*Xgwm635*	100	*Dn8*	PI 294994	[87]

# Xgwm44_182_ and Xgwm111_205_ are considered characteristic or functional fragments. See [60] for discussion. **, ***: Significant at *p* = 0.01 and *p* = 0.001, respectively.

**Table 2:** Rank of test entries using the *t*-distribution test (*p* = 0.05) of the mean damage rating (SEM) of each accession to biotype RWASA2.

Accessions Ranked from Most Resistant to Least Resistant	Mean RWASA2 Damage Rating (SEM) of Five Individual Example Plants of Each Accession	Postulated Potential Gene(s) in the Accession
PI 137739“S”	3.0 (0)	*Dn1*
CItr 2401	3.2 (0.5)	*Dn2401*
T06/16	3.2 (0.4)	*Dn1*, *Dn5*, *Dn8*, *Dn9*, *DnUnknown*
PI 586954	3.4 (0.5)	*Dnx*
PI 47545	3.8 (0.4)	*Dn47545*
PAN 3144	4.0 (0)	Gene not known
PI 626580	5.0 (1.1)	*Dn626580*
PI 586955	5.2 (1.9)	*Dnx*
T06/13	5.8 (2.7)	*Dn5*, *Dn8*, *Dn9*, *DnUnknown*
PI 243781	6.2 (2.6)	*Dn6*
PI 294994	6.8 (2.3)	*Dn5*, *Dn8*, *Dn9*, *DnUnknown*
T03/17	7.6 (2.2)	*Dn1*, *Dn2*
T05/02	7.8 (0.4)	*Dn5*, *Dn8*, *Dn9*, *DnUnknown*
PI 262660	8.0 (0.6)	*Dn2*
TugelaDn2	8.2 (0.4)	*Dn2*
Yumar	8.2 (0.7)	*Dn4*
BW991306	8.4 (0.8)	*Dn2401*, *Dn5*, *Dn8*, *Dn9*, *DnUnknown*
BW991405	8.4 (0.5)	*Dn2401*, *Dn5*, *Dn8*, *Dn9*, *DnUnknown*
PI 634775	8.5 (0.9)	*Dn8*
RIL-A50	8.6 (0.5)	*Dn2401*
Tugela-DN	8.8 (0.4)	*Dn1*
Betta-DN	9.0 (0)	*Dn1*
Gariep	9.0 (0)	*Dn1*
BettaDn2	9.0 (0)	*Dn2*
Hugenoot	9.0 (0)	Susceptible control
PI 634770	9.2 (0.4)	*Dn9*

**Table 3:** Accession (Sample name), *D. noxia* damage score (RWASA2) and marker haplotype for single plant examples screened with markers *Xgwm44*, *Xgwm111* and *Xgwm437.*

Accession (Sample Name)	Single Example Plant RWASA2 Score	Xgwm44	Xgwm111	Xgwm437
Betta-DN_1	9	120; 190	135; 210	120
Betta-DN_2	9	120; 190	135; 210	120
Betta-DN_3	9	120; 190	135; 210	120
Betta-DN_4	9	120; 190	135; 210	120
Betta-DN_5	9	120; 200	135; 220	120
Gariep_1	9	120; 190	135; 210	115
Gariep_2	9	120; 190	135; 210	115
Gariep_3	9	120; 190	135; 210	115
Gariep_4	9	120; 190	135; 210	115
Tugela-DN (V4483)	9	120; 190	135; 210	115
Tugela-DN (V4484)	9	120; 190	135; 210	115
Tugela-DN (V4485)	9	120; 190	135; 210	115
Tugela-DN (V4486)	9	120; 190	135; 210	115
Tugela-DN (V4487)	8	120; 190	135; 210	115
BettaDn2 (V4493)	9	120; 150; 190	135; 210	100
BettaDn2 (V4494)	9	120; 150; 190	135; 210	100
BettaDn2 (V4495)	9	120; 150; 190	135; 210	100
BettaDn2 (V4496)	9	120; 150; 190	135; 210	100
BettaDn2 (V4497)	-	120; 150; 190	135; 210	100
TugelaDn2 (V4578)	9	Null	135; 220	115
TugelaDn2 (V4579)	8	120; 150; 190	135; 210	100
TugelaDn2 (V4580)	8	120; 150; 190	135; 210	100
TugelaDn2 (V4581)	8	120; 150; 190	135; 220	115
TugelaDn2 (V4582)	8	120; 150; 190	135; 220	115
T05/02 (V4553)	7	Null	135; 210	100
T05/02 (V4554)	8	120; 175	135; 200	100
T06/13 (V4543)	8	120; 185	135; 200	120
T06/13 (V4544)	8	120; 185	135; 200	120
T06/13 (V4546)	3	120; 175	135; 200	120
T06/13 (V4547)	2	120; 175	135; 200	120
T03/17 (V4548)	7	120; 175	130; 200	120
T03/17 (4549)	9	120; 175	130; 200	120
T03/17 (V4550)	7	120; 175	130; 200	120
T03/17 (V4551)	7	120; 175	130; 200	120
T03/17 (V4552)	8	120; 175	130; 200	120
T06/16 (V4538)	4	120; 190	130; 200	135
T06/16 (V4539)	3	120; 175	130; 200	135
T06/16 (V4540)	3	120; 175	130; 200	135
T06/16 (V4541)	3	120; 175	130; 200	135
T06/16 (V4542)	3	120; 175	130; 200	135
PAN 3144_1	4	120; 190	135; 200	120
PAN 3144_2	4	120; 190	135; 200	120
PAN 3144_3	4	120; 195	135; 210	120
PAN 3144_4	4	120; 190	135; 200	120
PAN 3144_5	4	120; 195	135; 210	120

**Table 4:** Biotype(s) used for selection and/or development of wheat accessions, for the linkage analysis phenotyping and 7D marker alleles reported in the literature.

Accession(s) Selected or Developed with *D. noxia* Biotype	*D. noxia* Biotype Used to Phenotype for Linkage Analysis ₸	7D Markers Found (Reference)
PI 137739, PI 262660, PI 294994 selected with RWASA1 * and Betta-Dn1, Betta-Dn2, Betta-Dn9, Tugela-Dn1, Tugela-Dn2, Karee-Dn2, Karee-Dn8 developed with RWASA1 * [124]	RWA1 *	*Xgwm111 _200_*_,*210*,*220*_ [80] *Xgwm635 _100_* [80]
Sando selection 4040 × PI 220127 F_2:3_ developed with RWA1 [80]	RWA1	*Xgwm111 _225_* [86]
Carson x PI 262660 F_2:3_ developed with RWA1 [79]	RWA1	*Xgwm437_100_*_, *102*, *104*_ [79]
PI 372129, PI 243781, Thunderbird × PI 372129 (*Dn4*), Wichita × PI 372129 (*Dn4*), Wichita × PI 243781 (*Dn6*), and AL359 × PI 243781 (*Dn6*) developed with RWA1 [60]	RWA1	*Xgwm44_180_* [60] *Xgwm111_200_* [60]
F2 Betta-Dn1 †/Tugela-Dn2 † F2 Betta-Dn5 †/Tugela-Dn1 † F2 Karee-Dn5 †/Tugela-Dn2 † F2 PI 220127 (*Dnx*)/Tugela-Dn1 † F2 PI 220127 (*Dnx*)/Tugela-Dn2 † F2 PI 243781 (*Dn6*)/PI 137739(*Dn1*) # F2 PI 243781 (*Dn6*)/PI 372129(*Dn4*) # TC1 F1 Wichita//(Betta-Dn1 †/Tugela-Dn2 †) # TC1 F1 Wichita//(Karee-Dn5 †/Tugela-Dn2 †) TC1 F1 Wichita//(PI 243781 *Dn6*/PI 137739 *Dn1*) #	RWA1	*Xgwm44_180_*_, 200_ [86] *Xgwm111*_210_ [86]
NIL 92RL28, (PI 294994/5 * ‘Palmiet’) developed with RWASA1 *	RWASA1	*Xgwm44_180_* [87] *Xgwm111_215_* [87] *Xgwm437_105_* [87]
PI626580 × Yuma F_2:3_ developed with RWA2 [49]	RWA2	*Xbarc214_237_* [49] *Xgwm437*_124_ [49] *Xgwm473_244_* [49] *MS*1_251_ [49]
‘Glupro’ × CItr2401 F_2:3_ and CItr2401 × ‘Glupro’ F_2:3_ developed with RWA2 [95]	RWA2	*Xgwm111*_274_ [95] *Xgwm473*_244_ [95]
Tugela−Dn2, Tugela−Dn5, Palmiet−Dn5, PI 137739 (=SA1684), PI 262660 (=SA2199), PI 294,994 (=SA463), Chinese Spring 7DS dt, Chinese Spring 7DL dt, Tugela, Tugela × Tugela-Dn1 F_3:4_ developed with RWASA1 [83]	RWASA1	*Xgwm111 _210_*_, *240*, *250*_ [83]
134 diverse wheat accessions selected with RWASY [98]	RWASY	*wPt-733729* [98] *wPt-665471* [98] *wPt-3018* [98] *wPt-3291* [98]
DH mapping population derived from EGA Gregory × PI94365 developed without phenotyping [99]	RWASA1 RWASA2 RWASA3 RWASY RWATR	§ *QTL_RWASA1_7D* [99] *QTL_RWASA2_7D* [99] *QTL_RWATR_rolling_7D*[99]

₸ RWASA1 = Original South African biotype; RWASA2 = second South African Biotype; RWASA3 = third South African biotype; RWA1 = Original USA biotype; RWA2 = second USA biotype; RWASY = Original Syrian biotype; RWATR = Original Turkish biotype. † Initial identification and development of near-isogenic-lines with RWASA1 [124], further development with RWA1 [80,125]. # developed with RWA1. Inferred as original USA *D. noxia* biotype based on year of study. * Inferred as original South African *D. noxia* biotype based on year of study. § Author original designation modified as follows to reflect common RWA biotype nomenclature: *‘QTL_RWA SAB1_7D*’ presented as ‘*QTL_RWASA1_7D’*; ‘*QTL_RWA_SAB2_7D*’ presented as ‘*QTL_RWASA2_7D*’; ‘*QTL_RWA_Trolling_7D*’ presented as ‘*QTL_RWATR_rolling_7D*’.

**Table 5:** Study panel of wheat accessions, their pedigree, accession status, postulated *D. noxia* gene information, and customary mean resistance reaction (SEM) to RWASA1 and RWASA2.

Wheat Accession	Pedigree	Accession Status	*D. noxia* R-Gene(s) Potentially Present	Mean (SEM) RWASA1 Score *	Mean (SEM) RWASA2 Score *
Hugenoot	Betta//Flamink/Amigo	Cultivar, Susceptible check	None	9.3 (0.45)	9.0 (0.58)
PI 137739”S”	Not applicable	Selection from *Dn1 D. noxia* R-donor, Landrace *ex*. Iran	*Dn1 and/or Dn137739”S”*	5.1 (1.68)	4.5 (1.94)
Betta-DN	PI 137739/*4Betta(4)	Cultivar	*Dn1*	5.5 (1.74)	8.2 (1.09)
Gariep	PI 137739/*4 Molopo(20)	Cultivar, Differential check	*Dn1*	5.3 (0.55)	8.0 (1.01)
Tugela-DN	Tugela*4/PI 137739	Cultivar	*Dn1*	5.4 (1.34)	7.7 (0.98)
PI 262660	Not applicable	*D. noxia* R-donor, Landrace *ex*. Azerbaijan	*Dn2*	4.4 (0.54)	6.7 (2.20)
BettaDn2	Betta*4/PI 262660	Advanced breeding-line [SYN = PI 634769]	*Dn2*	5.3 (1.07)	-
TugelaDn2	Tugela*4/PI 262660	Advanced breeding-line [SYN = PI 634772]	*Dn2*	6.0 (1.18)	-
Yumar	Yuma/PI-372129//CO-850034/3/4*Yuma	Cultivar, Differential check	*Dn4*	5.9 (1.36)	7.6 (1.82)
PI 294994	Not applicable	*D. noxia* R-donor, Landrace *ex*. Bulgaria	*Dn5*, *Dn8*, *Dn9*, *DnUnknown*	4.0 (0.80)	4.1 (0.25)
T05/02	PI-294994/*4Molen	Advanced breeding-line	*Dn5*, *Dn8*, *Dn9*, *DnUnknown*	3.9 (1.21)	3.9 (0.69)
T06/13	Karee/4/PI-294994/*4Gamtoos/3/YD”S”/BON//Dove”S” #	Advanced breeding-line	*Dn5*, *Dn8*, *Dn9*, *DnUnknown*	3.9 (1.74)	3.7 (0.92)
PAN 3144	PANNAR ^®^ Proprietary information	Cultivar, Differential check	Gene not known	4.1 (0.80)	3.5 (0.59)
PI 243781	Not applicable	*D. noxia* R-donor, Landrace *ex*. Iran	*Dn6*	3.1 (0.87)	5.5 (2.02)
PI 634775	Karee*6/PI 294994	Advanced breeding-line	*Dn8*	8.1 (1.92)	-
PI 634770	PI 294994/*4Betta	Advanced breeding-line	*Dn9*	5.6 (0.66)	-
PI 586954 [KS94WGRC29]	PI-220127/P5//TAM200/KS87H66	Advanced breeding-line	*Dnx*	4.4 (0.75)	4.1 (0.46)
PI 586,955 [KS94WGRC30]	PI-220127/P5//TAM200/KS87H66	Advanced breeding-line	*Dnx*	3.2 (1.05)	4.1 (1.42)
PI 047545	Not applicable	*D. noxia* R-donor, Landrace *ex*. Iran	*Dn47545*	3.2 (1.49)	3.7 (0.74)
PI 626580	Not applicable	*D. noxia* R-donor, Landrace *ex*. Iran	*Dn626580*	5.1 (1.35)	4.5 (1.31)
CItr 2401	Not applicable	*D. noxia* R-donor, Resistant check, Landrace *ex*. Tajikistan	*Dn2401*	3.6 (0.58)	4.0 (0.58)
RIL-A50	Kavkaz*5/CItr 2401	F_6_ recombinant inbred line	None	8.0 (1.65)	6.6 (1.76)
T03/17	SST333^(*ex.*PI262660)//^661L1–33/Tugela-DN^(*ex.* PI 137739)^	Advanced breeding-line	*Dn1 + Dn2*	4.4 (1.11)	5.1 (1.20)
T06/16	Gariep^(*ex*.PI137739)^/4/PI-294994/*4Gamtoos/3/YD”S”/BON//Dove”S”	Advanced breeding-line	*Dn1 + Dn5*, *Dn8*, *Dn9*, *Dn*U*nknown*	4.1 (1.93)	3.3 (0.94)
BW991405	PI-294994/*4BTA//TMP/CI13523-STW646408/4/FKS*3/3/W66136//Mayo/WRR4255-49-5/5/CItr 2401/*4Kariega	Advanced breeding-line	*Dn2401 + Dn5*, *Dn8*, *Dn9*, *DnUnknown*	7.0 (1.49)	6.4 (1.80)
BW991308	PI-294994/4*Molen//CItr 2401/*4Kariega	Advanced breeding-line	*Dn2401 + Dn5*, *Dn8*, *Dn9*, *DnUnknown*	-	4.9 (2.16)

* Scores based on visual *D. noxia* damage to seedlings which is rated from 1 to 10 where 1 = Small isolated chlorotic spots, 2 = Small chlorotic spots, 3 = Chlorotic spots in rows, 4 = Chlorotic splotches, 5 = Mild chlorotic streaks, 6 = Prominent chlorotic streaks, 7 = Severe streaks, leaves fold conduplicate, 8 = Severe streaks, leaves roll convolute, 9 = Severe streaks, leaves roll tightly, and 10 = Plant dying [16]. Means collated from multiple prior evaluations with *n* ≥ 11 ≤ 40 (Supplementary Table S2). # Note 1: Gamtoos = Veery#3 [140,141,142] is a susceptible cultivar with the 1B/1R translocation released in South Africa in 1983. Multiple resistant accessions were developed from it by ARC-Small Grain Centre, Bethlehem, South Africa, namely Gamtoos-DN (*Dn1*) [143] GamtoosDn2 and GamtoosDn5 [144] and Stellenbosch University, Stellenbosch, RSA, ‘GamtoosDn7′ [142,143].

**Table 6:** Susceptible, differential and resistant checks used in the study, the *D. noxia* R-genes they reportedly carry and reactions to four South African *D. noxia* biotypes (Adapted from [145]). A typical damage rating score of 1–3 is considered highly resistant (HR); 4, 5 is resistant (R); 6, 7 is moderately resistant (MR) and 8–10 is susceptible (S).

Differential Checks	*D. noxia* R-Gene	RWASA1	RWASA2	RWASA3	RWASA4
Hugenoot	*None*	S	S	S	S
Gariep	*Dn1*	MR	S	S	S
Yumar	*Dn4*	MR	MR	S	S
PAN 3144	*Gene not known*	R	R	R	S
CItr 2401	*Dn2401*	R	R	R	R

## Supplementary Materials

Supplementary Materials can be found at https://www.mdpi.com/1422-0067/21/21/8271/s1. Figure S1: Map of South African wheat production regions. Table S1: Genotypic data of individual sample plants including PIC values and orthologs of markers *Xgwm44*, *Xgwm111* and *Xgwm437*. Table S2: Historic data used to calculate the mean resistance reaction (SEM) to RWASA1 and RWASA2 for the test panel accessions.

## Abbreviations

ICARDA	International Center for Agricultural Research in the Dry Areas
PIC	Polymorphism Information Content
QTL	Quantitative Trait Loci
RWA1	Russian wheat aphid, United States of America, original biotype
RWASA1	Russian wheat aphid, South Africa, Original biotype
RWASA2	Russian wheat aphid, South Africa, biotype 2
RWASA3	Russian wheat aphid, South Africa, biotype 3
RWASA4	Russian wheat aphid, South Africa, biotype 4
RWASA5	Russian wheat aphid, South Africa, biotype 5
RWASY	Russian wheat aphid, Syria, original biotype
RWATR	Russian wheat aphid, Turkey, original biotype
SSR	Single Sequence Repeat
