# Supplementary figures and images for: Elusive Diagnostic Markers for Russian Wheat Aphid Resistance in Bread Wheat: Deliberating and Reviewing the Status Quo
Source: Int J Mol Sci. 2020 Nov 4;21(21):8271. doi: 10.3390/ijms21218271 (PMC7663459; doi:10.3390/ijms21218271)

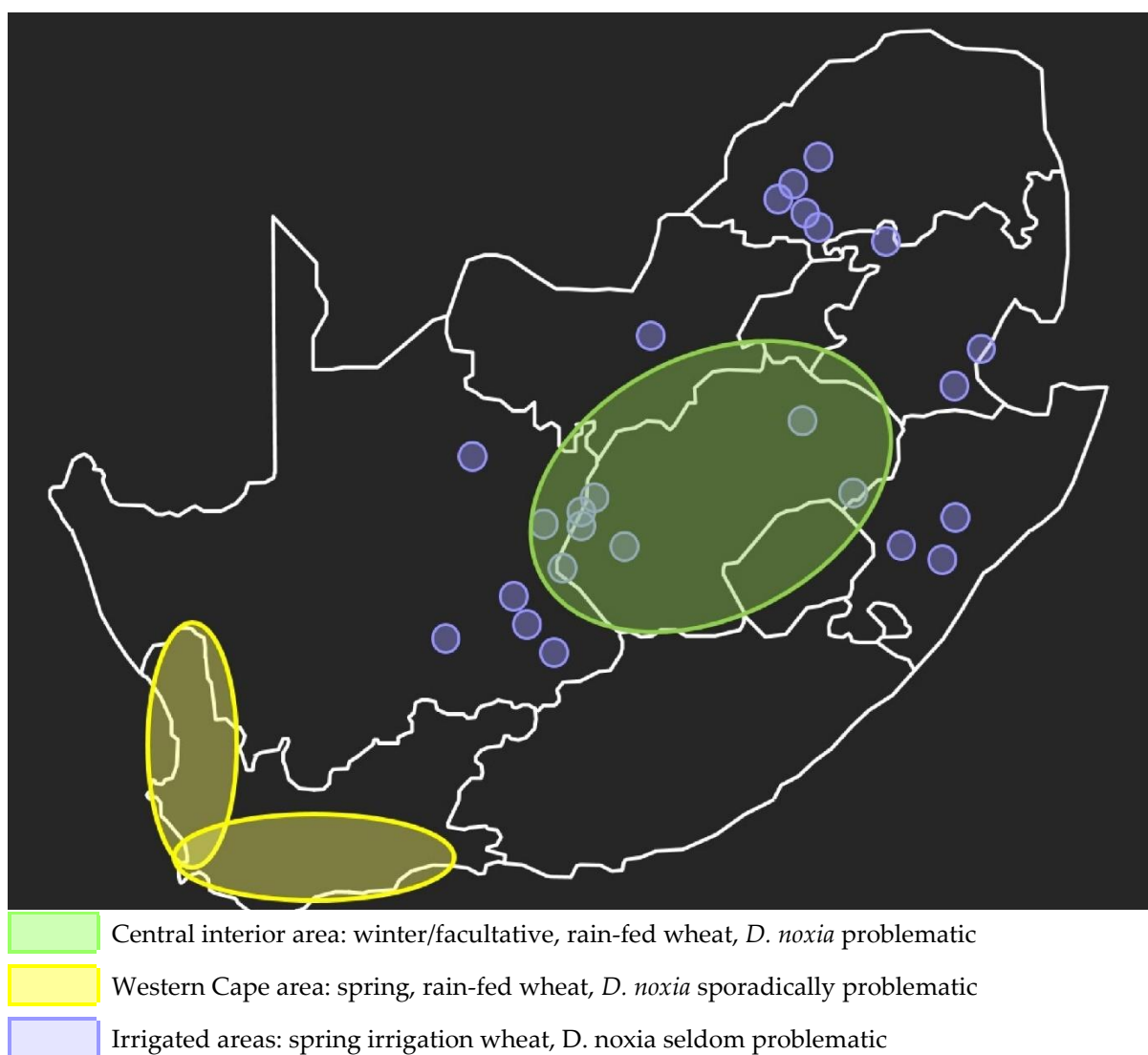

Figure A1: Map of wheat production areas in South Africa.

Supplement: Supplementary file 1 [file ijms-21-08271-s001.zip › ijms-905923 SI/Supplementary files/20200731 New submission - Revised IJMS-763731 Supplementary1 Figure A1.pdf]
